# Antimicrobial susceptibility pattern of *Staphylococcus aureus* isolates from clinical specimens at Kenyatta National Hospital

**DOI:** 10.1186/s13104-018-3337-2

**Published:** 2018-04-03

**Authors:** Wilfred Gitau, Moses Masika, Moses Musyoki, Beatrice Museve, Titus Mutwiri

**Affiliations:** 1 Department of Medical Microbiology, University of Nairobi, P.O Box 19676-00202, Nairobi, Kenya; 2 Department of Laboratory Medicine, Microbiology, Kenyatta National Hospital, P.O Box 20723-00202, Nairobi, Kenya; 3 Department of Medical Laboratory, Kenya Methodist University, P.O Box 45240-00100, Nairobi, Kenya

**Keywords:** Methicillin resistant *Staphylococcus aureus*, Drug resistance, Susceptibility, *Staphylococcus aureus*, Vancomycin, Kenyatta National Hospital

## Abstract

**Objective:**

To determine antibiotic susceptibility pattern of *S. aureus* isolates from clinical specimens collected from patients at Kenyatta National Hospital from March 2014–February 2016, and to determine the prevalence and quarterly trends of MRSA throughout the study period.

**Results:**

A total of 944 *S. aureus* isolates were analyzed. High sensitivity of *S. aureus* was observed for quinupristin/dalfopristin (100%), tigecycline (98.2), imipenem (98%), nitrofurantoin (97.6%), linezolid (97.3%), teicoplanin (97.1%) and vancomycin (95.1%). High resistance was recorded against penicillin G (91.9%), trimethoprim/sulfamethoxazole (56.9%) and tetracycline (33.2%). MRSA prevalence among the patients at KNH was 27.8%. Highest proportion (80%) of MRSA was in burns unit. Both MRSA and MSSA were highly susceptible to quinupristin/dalfopristin, tigecycline, linezolid, nitrofurantoin, ampicillin/sulbactam and vancomycin and showed high resistance to commonly used antibiotics such as gentamycin, erythromycin, levofloxacin and tetracycline. A majority of isolates were from pus specimen (68%).

## Introduction

*Staphylococcus aureus* is a Gram-positive bacterium living as a commensal on the skin, mouth and upper respiratory system, making it a risk factor for opportunistic and nosocomial infections. Resistance to commonly used antimicrobial drugs is frequently encountered with *S. aureus.* Some of the mechanisms in resistance include; inactivation of antibiotics by the enzymes, decreased affinity for the antibiotics caused by alteration of the target, efflux pumps, and trapping of the antibiotic [1].

*Staphylococcus aureus* causes skin, bone, soft tissue infections, urinary tract infections, pneumonia, healthcare-associated bacteremia in community and hospital settings and other invasive infections. Multidrug-resistant strains particularly Multidrug Resistant *Staphylococcus aureus* (MRSA) strains are common causes of nosocomial infections and are associated with increased morbidity and mortality [2, 3].

Report from the National Nosocomial Infections Surveillance System of the Centers for Disease Control and Prevention, (2013–2015) showed that MRSA in India and USA accounts for > 60% of *S. aureus* isolates causing nosocomial infection in ICUs [4].

There is no conclusive local data on the magnitude of multidrug resistance MRSA infection burden in Kenyan hospitals. In study done from patients in select hospitals in Nairobi, MRSA infections were mostly isolated at public healthcare facilities serving economically disadvantaged Nairobi’s population, like those living in urban informal settlements [5].

Another study done in Kenya showed there was high number of genetically indistinguishable isolates, which suggested there was local transmission of MSSA and MRSA [6].

Multidrug-resistant strains limit the therapeutic options, creating an economic and social burden to the healthcare system. Horizontal gene transfer in the hospital setting is responsible for disseminating antibiotic resistant determinants. Chromosomal mutation antibiotics selection is also responsible for antibiotics resistance.

The aim of this study was to evaluate the antimicrobial susceptibility pattern of *Staphylococcus aureus* at KNH from April 2014 to February 2016. Previous studies done in Kenya were targeting specific locations in the hospital and were within short periods of time. This study gives a broader picture of the susceptibility pattern of *S aureus* in different locations in 3 years.

## Main text

### Methodology

This was a retrospective study based on electronic laboratory records of *Staphylococcus aureus* isolates from clinical specimens analyzed at KNH microbiology laboratory.

Data was retrieved from VITEK-2 Antibiotic Susceptibility Testing System, imported to WHONET software through BACLINK (World Health Organization). Analysis was done using WHONET and IBM SPSS V.21. VITEK 2 Gram Positive identification card (bioMerieux) was used to identify *S. aureus* sub-species *aureus*. All the isolates had an ID confidence of excellent identification with an average percent probability of 96%.

Methicillin resistance was determined using cefoxitin screening.

All isolates were from patients’ clinical specimens (mainly pus, urine, blood and tracheal aspirates) and were analyzed according to the 2015 Clinical & Laboratory Standards Institute (CLSI M100-S25) standards.

Antibiotics tested against *S. aureus* include penicillin G (10 units), oxacillin (30 µg cefoxitin), gentamycin (10 µg), fusidic acid (10 µg), cefuroxime (30 µg), cefuroxime axetil (30 µg), imipenem (10 µg), tobramycin (10 µg), rifampicin (5 µg), levofloxacin (5 µg), clindamycin (2 µg), trimethoprim/sulfamethoxazole (1.25/23.75 µg), moxifloxacin (5 µg), nitrofurantoin (300 µg), linezolid (30 µg), vancomycin (30 µg), teicoplanin (30 µg), quinupristin/dalfopristin (15 µg), tetracycline (30 µg), tigecycline (15 µg), erythromycin (15 µg) and ampicillin/sulbactam (10/10 µg).

The sample size was calculated using Fishers formula [7]

$$\begin{aligned} {\text{n}} =\, & {\text{z}}^{2} {\text{pq}}(1 - {\text{q}})/{\text{d}}^{2} \\ {\text{N}} =\, & 1.96^{2} \times 0.47(1 - 0.47)/0.05^{2} = 382. \\ \end{aligned}$$

Univariate analysis was done using frequency distributions and proportions for categorical variance such as antimicrobial resistance and gender.

Bivariate analysis was done by use of Chi square to assess for association between categorical variables such as susceptibility of antibiotics and infections location. Data was presented in tables and graphs.

### Results

A total of 944 *S. aureus* isolates were analyzed, 33% (311/944) of pathogens were isolated in 2014, 62% (586/944) in 2015 and 5% (47/944) in 2016. Majority of the analyzed isolates, 54% (511/944), were from male patients.

Internal Medicine department recorded the highest number of isolates, 187/944 (20%). A majority of the isolates were from pus specimen, 638/944 (68%), tracheal aspirate (15%) and blood (11%). Other specimen types, 26/944 (3%) included tissue, sputum, eye, throat, CSF and the unindicated specimens.

High susceptibility was seen with quinupristin/dalfopristin (100%). High resistance was observed with penicillin G (92%) and trimethoprim/sulfamethoxazole (57%) (Table 1).

**Table 1:** Antimicrobial susceptibility profile

Antibiotic	No. of isolates	%S	%NS
Quinupristin/dalfopristin	98	100	0
Ampicillin/sulbactam	98	98	2
Imipenem	98	98	2
Nitrofurantoin	929	98	2
Tigecycline	925	98	2
Linezolid	929	97	3
Teicoplanin	929	97	3
Fosfomycin	791	96	4
Vancomycin	927	95	5
Clindamycin	928	93	7
Rifampin	830	92	8
Fusidic acid	791	88	12
Gentamicin	831	87	13
Tobramycin	831	87	13
Cefuroxime	98	85	15
Cefuroxime axetil	98	85	15
Mupirocin	831	82	18
Levofloxacin	929	78	22
Moxifloxacin	929	78	22
Erythromycin	930	73	26
Cefoxitin screen	831	72	28
Oxacillin	828	71	29
Tetracycline	929	67	33
Trimethoprim/sulfamethoxazole	929	43	57
Penicillin G	829	8	92

*S* Susceptible; *NS* non susceptible (resistant and intermediate)

Isolates from pus to HVS specimens showed high susceptibility to ampicillin-sulbactam (100%) while isolates from blood showed least susceptibility (90%) Isolates from pus specimen recorded high resistance to cephalosporins (17%). Isolates from all specimens showed high susceptibility to imipenem.

*Staphylococcus aureus* isolates from HVS showed high resistance to tobramycin (16%) and gentamycin (24%), but showed high susceptibility to quinupristin/dalfopristin (100%), cefuroxime (100%) and imipenem (100%) High susceptibility to quinolones was observed in isolates from other specimens (90%) and low susceptibility was observed in isolates from tracheal aspirates (75%).

As shown in Table 1, isolates from all the specimens showed 100% sensitivity to quinupristin/dalfopristin. Isolates from HVS showed high resistance to tetracycline (41%), erythromycin (34%) and clindamycin (19%), no resistance was recorded from isolates from CSF to clindamycin.

Nine per cent of isolates from tracheal aspirates recorded complete resistance to vancomycin (VRSA) and 3% recorded intermediate resistance to vancomycin (VISA). Isolates from tracheal aspirates also showed lower susceptibility to teicoplanin (93%). *S. aureus* isolates from CSF (100%) and pus (99%) recorded high susceptibility to tigecycline.

Isolates from pus specimens recorded high resistance to trimethoprim/sulfamethoxazole (60%). High resistance to linezolid was observed in isolates from tracheal aspirates (7%). Isolates from HVS recorded high resistance to fosfomycin (24%), mupirocin (36%) and nitrofurantoin (6%).

### MRSA distribution by year

The overall prevalence of MRSA was 27.8%. MRSA prevalence for 2014 was 34.9% (103/298) and 2015 recorded 25.8% (124/481). 2016 had MRSA prevalence of 21.7% representing samples for 2 months (January and February). There was no significant difference between MRSA isolation and the year of isolation (P = 0.159).

### MRSA Distribution by specimen

The highest prevalence of MRSA was observed in pus specimen 153/232 (66%).

### Quarterly trend of MRSA

**Fig. 1:**
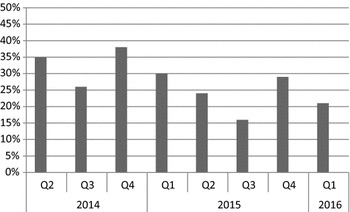
Quarterly trend of MRSA (No. 944). *Q* Quarter

A high proportion of MRSA, 48/127 (38%), was observed in the fourth quarter (Q4) of 2014 as shown in Fig. 1. Least proportion, 18/110 (16%) was observed in the third quarter of 2015 (Fig. 1).

### Susceptibility of MRSA to different antibiotics

High susceptibility of MRSA isolates was observed with tigecycline (97%), nitrofurantoin (96%), linezolid (95.3%), teicoplanin (94.8%) and vancomycin (94.7%). This is shown in Table 2.

**Table 2:** Susceptibility of MRSA to different antibiotics

Antibiotic	No. of MRSA	% Susceptible
Tigecycline	230	97
Nitrofurantoin	231	96
Linezolid	232	95
Teicoplanin	229	95
Vancomycin	228	95
Fosfomycin	220	92
Clindamycin	221	86
Rifampicin	230	81
Tobramycin	219	73
Fusidic acid	220	72
Gentamycin	218	66
Moxifloxacin	174	58
Mupirocin	231	56
Levofloxacin	228	44
Erythromycin	205	37
Tetracycline	231	35
Trimethoprim/sulfamethoxazole	232	25
Oxacillin	229	25
Penicillin G	229	3

### Discussion

The aim of this study was to evaluate the antimicrobial susceptibility pattern of *S. aureus* from clinical specimens at Kenyatta National Hospital. In our study, most *S. aureus* strains (68%) were isolated from pus specimen. This is consistent with a previous study done at KNH [8]. In a study done to determine the antimicrobial susceptibility pattern of *S. aureus* strains isolated from hospitalized patients in Iran, most of the isolates were from blood specimens (29%) [9]. Another study done on prevalence and antibiotic susceptibility pattern of *S. aureus* from clinical isolates in Nigeria showed a majority of the isolates were from urine specimens (76%) [10].

The high number of *S. aureus* isolated in pus may be attributed to exposure of wounds which makes them more prone to infections and poor hygiene.

*Staphylococcus aureus* isolates showed high sensitivity to quinupristin/dalfopristin, imipenem, nitrofurantoin, tigecycline, ampicillin/sulbactam and linezolid. This is consistent with a similar study done in Iran [11]. Research done on antibiotics currently used in the treatment of infections caused by *S. aureus* in Australia indicates quinupristin/dalfopristin and linezolid have good antistaphylococcal activity but are very expensive [12].

In our study, high resistance of *S. aureus* isolates was observed against penicillin G (91.9%), and trimethoprim/sulfamethoxazole (56.9%). This finding is similar to those of studies done at KNH [13], in Eritrea [14], in Nigeria [15], and Namibia [16]. This resistance could be attributed to the mechanism of resistance like the permeability barrier, efflux pumps, mutational or recombinational changes in the target enzymes and acquired resistance by drug-resistant target enzymes in trimethoprim/sulfamethoxazole and alteration of the target with decreased affinity for the antibiotic in penicillin [1].

In this study, MRSA was tested using cefoxitin screening. Overall MRSA prevalence was 27.8%. This prevalence was lower than in previous studies that reported 46.5% of MRSA prevalence at KNH [13], 31.5% in Uganda [17] and 46.3% in Iran [18].

This difference could be attributable to interventions that may have been effected during the study period such as infection control and improved antimicrobial stewardship and/or appropriate antibiotic use, this difference in MRSA prevalence could also be attributed to the different laboratory techniques used to correctly identify MRSA. KNH started using automated VITEK-2 system in mid 2013. VITEK^@^ 2 Gram Positive identification card (vitek^@^2 gp card bioMerieux) used at KNH is sensitive and specific to subspecies *S. aureus aureus* [18, 19].

In contrast, MRSA prevalence was lower in studies done in two private hospitals in Nairobi, Kenya, which showed a 3.8% prevalence [20]; a study in Eritrea that recorded 9% prevalence and 0.03% prevalence in Dutch hospitals [13, 21]. The low prevalence of MRSA in private hospitals could be attributed to better infection controls. This shows there is high variance of MRSA prevalence from different countries. Majority of MRSA was isolated from pus specimens, 154/232 (66%). Our finding concurs with studies done in two private hospitals in Nairobi, a Namibian institute of pathology and a tertiary health institution in Nigeria [14, 15, 22]. In contrast, other studies done in Nigeria, Iran and Jamaica showed different specimens were predominant [9, 23, 24]. The high number of MRSA from pus in our study could be due to exposure of wounds and abscesses to *S. aureus*. Carriage of *S. aureus* on the skin makes wounds more prone to MRSA infections.

In this study, 5% of MRSA isolates were resistant to vancomycin. This finding is similar to a study done in Iran which showed 5% of the MRSA isolates were resistant to vancomycin [11]. This contrasts similar studies done in a tertiary care hospital in India and pediatrics and neonatal intensive care patients at KNH which, respectively showed 3.5 and 1% resistance to vancomycin among MRSA [12, 23]. Studies done on antimicrobial susceptibility of MRSA in hospitalized patients in Iran, two hospitals in India and two private hospitals in Kenya showed 100% susceptibility to vancomycin [8, 22, 25]. Our study showed *S. aureus* isolates were highly susceptible to newer drugs. These drugs include; quinupristin/dalfopristin, tigecycline, imipenem, teicoplanin, vancomycin and linezolid. Similar studies done in Kenya and USA have shown *S. aureus* to be highly susceptible to ceftobiprole, tigecycline, linezolid, teicoplanin, vancomycin and daptomycin [22, 26].

This finding differs from a study done by Arianpoor et al. on antimicrobial susceptibility pattern of *S. aureus* isolates against newly marketed antibiotics in Iran which showed 5.5% of MRSA isolates were resistant to linezolid, 5.9% of to quinupristin–dalfopristin and 18.9% to tigecycline [11].

### Conclusion and recommendations

Our study showed that burns unit had the highest (80%) proportion of MRSA isolates. Both MRSA and MSSA were highly susceptible to quinupristin/dalfopristin, tigecycline, linezolid, nitrofurantoin, ampicillin/sulbactam, and vancomycin, but showed high resistance to commonly used antibiotics such as gentamycin, erythromycin, levofloxacin, SMX-TMP and tetracycline. Infection control measures should be enhanced in burns unit. Information from this study may be used in future as a baseline for follow-up to the susceptibility trend of various drugs to be used for the treatment of *S. aureus* infections. Routine screening of MRSA and regular studies should be conducted to find out the sources of MRSA. It is important to do culture and sensitivity of relevant specimens when *S aureus* infection is suspected. There is need for further research on molecular studies evaluating the resistance genes and monitoring the epidemiology of multiple drug resistant *S. aureus* and MRSA.

## Limitations

This was a retrospective study, where some information such as specimen type, collection date, age of the patient, clinical information, previous antibiotics use, duration of patient stay in the hospital and outcome of the therapy were missing.

## Abbreviations

Bla: beta-lactamase
CA-MRSA: community associated methicillin resistant *Staphylococcus aureus*
CSF: cerebrospinal fluid
HA-MRSA: hospital associated methicillin resistant *Staphylococcus aureus*
ICU: intensive care unit
KNH: Kenyatta National Hospital
MRSA: methicillin resistant *Staphylococcus aureus*
NICU: neonatal intensive care unit
PBP: penicillin binding proteins
PICU: pediatric intensive care unit
SOP: standard operating procedure
UoN: University of Nairobi
VISA: vancomycin intermediate *Staphylococcus aureus*
VRSA: vancomycin resistant *Staphylococcus aureus*
WHO: World Health Organization
